# 

**Published:** 1889-01

**Authors:** 


CONTENTS.

Vol. 36 No. 1.

| | PAGE. |
|---|---|
| 1889 | 1 |
| Theosophy | 2 |
| "Pity 'Tis 'Tis True | 7 |
| What Shall Appeal to Women | 7 |
| Care of the Feet | 9 |
| How to Live Long | 9 |
| Rapidity of the Multiplication of Bacteria | 11 |
| Remarkable Memories | 11 |
| The Use of Tobacco | 12 |
| A Loss to Mental Science | 14 |
| Treatment of Rheumatism | 15 |
| Precautions in Bathing | 15 |
| The Vital Functions | 16 |
| Indigestion | 17 |
| Some Causes of Cancer | 18 |
| An Oculist's Test | 19 |
| Value of Rest | 19 |
| Book Notices | 20 |
| Household | 21 |
| Miscellaneous | 23 |
| "Poveri! Poveris!" | 24 |

INDEX TO ADVERTISEMENTS OF HALF A PAGE AND OVER.

| | PAGE. |
|---|---|
| Murdock's Liquid Food Co | I |
| Stevensville Mills | II |
| The Herb Cure Co | II |
| The Banner of Life and Home Physician | III |
| Hollow Suppositories | IV |
| Lactopeptine | V |
| The Family Cyclopædia of Useful Knowledge | VI |
| Revised Premium List | VIII |
| Rio Chemical Co | X |
| Davis' Elixir of Health | XI |

